# A novel phantom and procedure providing submillimeter accuracy in daily QA tests of accelerators used for stereotactic radiosurgery^*^

**DOI:** 10.1120/jacmp.v17i4.6295

**Published:** 2016-07-08

**Authors:** Ivan A. Brezovich, Richard A. Popple, Jun Duan, Sui Shen, Xingen Wu, Sidi Benhabib, Mi Huang, Rex A. Cardan

**Affiliations:** ^1^ Department of Radiation Oncology University of Alabama at Birmingham Birmingham AL USA

**Keywords:** stereotactic radiosurgery, quality assurance phantom, end‐to‐end test, alignment test

## Abstract

Stereotactic radiosurgery (SRS) places great demands on spatial accuracy. Steel BBs used as markers in quality assurance (QA) phantoms are clearly visible in MV and planar kV images, but artifacts compromise cone‐beam CT (CBCT) isocenter localization. The purpose of this work was to develop a QA phantom for measuring with sub‐mm accuracy isocenter congruence of planar kV, MV, and CBCT imaging systems and to design a practical QA procedure that includes daily Winston‐Lutz (WL) tests and does not require computer aid. The salient feature of the phantom (Universal Alignment Ball (UAB)) is a novel marker for precisely localizing isocenters of CBCT, planar kV, and MV beams. It consists of a 25.4 mm diameter sphere of polymethylmetacrylate (PMMA) containing a concentric 6.35 mm diameter tungsten carbide ball. The large density difference between PMMA and the polystyrene foam in which the PMMA sphere is embedded yields a sharp image of the sphere for accurate CBCT registration. The tungsten carbide ball serves in finding isocenter in planar kV and MV images and in doing WL tests. With the aid of the UAB, CBCT isocenter was located within 0.10±0.05 mm of its true positon, and MV isocenter was pinpointed in planar images to within 0.06±0.04 mm. In clinical morning QA tests extending over an 18 months period the UAB consistently yielded measurements with sub‐mm accuracy. The average distance between isocenter defined by orthogonal kV images and CBCT measured 0.16±0.12 mm. In WL tests the central ray of anterior beams defined by a $1.5\times 1.5\;{\text{cm}}^{2}$ MLC field agreed with CBCT isocenter within 0.03±0.14 mm in the lateral direction and within 0.10±0.19 mm in the longitudinal direction. Lateral MV beams approached CBCT isocenter within 0.00±0.11 mm in the vertical direction and within ‐0.14±0.15 mm longitudinally. It took therapists about 10 min to do the tests. The novel QA phantom allows pinpointing CBCT and MV isocenter positions to better than 0.2 mm, using visual image registration. Under CBCT guidance, MLC‐defined beams are deliverable with sub‐mm spatial accuracy. The QA procedure is practical for daily tests by therapists.

PACS number(s): 87.53.Ly, 87.56.Fc

## I. INTRODUCTION

MV portal images provide relatively poor definition^(1)^ and are being increasingly replaced by images from gantry‐mounted planar kV and CBCT systems. Considering the crucial role kV images are playing in patient setup, the AAPM TG‐142^(2)^ protocol recommends imaging and treatment system coordinate coincidence of ≤2 mm in nonstereotactic applications and ≤1 mm agreement if the accelerator is used for SRS or SBRT. Tests should be done daily. The more recent AAPM TG‐179 report also recommends daily tests and discusses the importance of couch motion accuracy.^(3)^ ACR‐AAPM and ACR‐ASTRO guidelines recommend 1 mm accuracy in delivery.^4^, ^5^

A number of quality assurance (QA) systems for assessing accelerator performance are available. Sub‐mm accuracy has been achieved by systems that integrate phantoms with image‐analysis software. These comprise custom made systems^6^, ^7^, ^8^, ^9^ and commercial systems. Commercial examples include the Quasar system (Modus Medical Systems, London, Ontario, Canada) and the ISO Cube (Computerized Imaging Reference Systems, Inc., Norfolk, VA), claiming “14mm accuracy” and “0.1 mm accuracy,” respectively. Linear accelerator vendors offer similar hardware/software tools for QA, such as the Isocal system provided by Varian (Varian Medical Systems, Palo Alto, CA).^(10)^ While impressive accuracy can be achieved with these systems, the custom software needed to use these tools obscures the measurements, making manual evaluation complicated. Furthermore, manual image registration using existing tools yields less accuracy. The Mimi phantom (Standard Imaging, Middleton, WI) can verify isocenter to within 1 mm according to the manufacturer. Similar accuracy has been reported for the image‐guided process using a cubic phantom containing 0.95 mm steel spheres.^(11)^ To the best of our knowledge, this paper is the first report on long‐term clinical evaluation of an independent QA phantom providing sub‐mm accuracy without requiring custom image‐analysis software.

## II. MATERIALS AND METHODS

**Figure 1:**
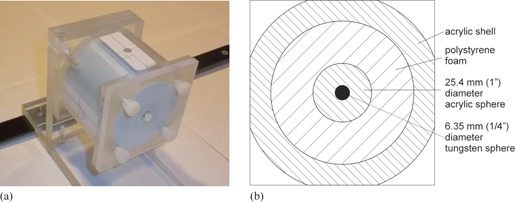
Universal Alignment Ball (UAB): (a) the short crosses drawn on the superior and lateral surfaces of the UAB are the offset marks for quick checks of laser alignment, while the long lines mark the center of the concentric balls; (b) cross‐sectional view.

When photographic film is used for position measurements, small BBs are appropriate markers since the high spatial resolution of film renders their locations with great accuracy. In digital images however, the position of a small object is known only to the extent of the pixel and CT slice where it is located. Since pixels of kV and MV images are about 0.4 mm or larger and CBCT slices are 1 mm thick, small BBs cannot provide one‐tenths‐mm accuracy. However, it has been demonstrated that with computer aid the position of regular‐shaped objects that extend over many pixels can be found with accuracy substantially better than pixel size.^9^, ^12^ The center of a sphere having a radius of about 10 times the pixel dimensions, e.g., can be determined with accuracy of one‐tenth pixel size.^(13)^ Hence, it should be possible to locate the center of the 12.7 mm (1/2”) radius polymethylmetacrylate (PMMA) sphere in the Universal Alignment Ball (UAB) in CBCT images with 0.4 mm pixel size and 1 mm slice thickness within 0.1 mm (Fig. 1). Likewise, the center of the 3.175 mm (1/8”) radius tungsten carbide ball should be detectable to about 0.04 mm in planar kV and MV images having 0.4 mm pixels.

**Figure 2:**
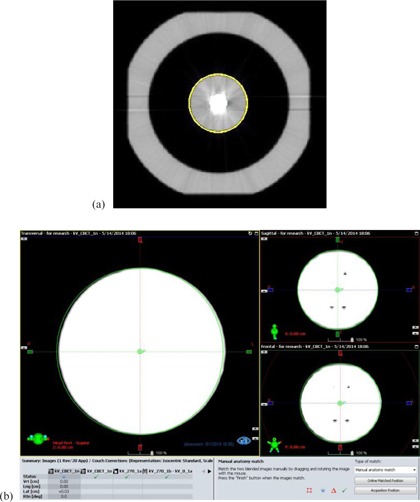
Visual registration procedure: (a) transverse CBCT image of the UAB; (b) a narrow display window (‐450 to ‐430 HU), yields a sharp outline of the PMMA sphere that is visually registered with a computer‐generated 25 mm–diameter circle in the three principal planes. For illustration purposes, the image is misaligned by 0.3 mm in the lateral direction. On a computer screen <0.1 mm mismatch is readily recognized.

**Figure 3:**
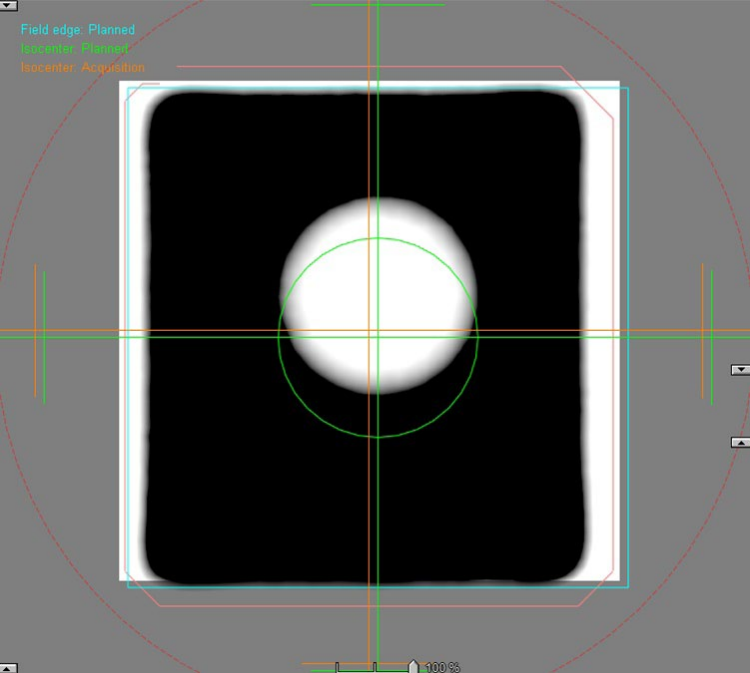
Winston‐Lutz test. After registration of the tungsten carbide ball with the circular outline (green color), the display window has been readjusted for registration of the superior and inferior borders of the MLC‐defined $1.5\times 1.5\;{\text{cm}}^{2}$ radiation field with the square contour (blue color). After readjustment of the display window, the lateral borders are registered. The red outlines are the field edges detected by the computer. The tick marks represent 1 cm.

In order to circumvent the need for computer aid, our QA procedure uses a narrow display window which produces sharp outlines of the PMMA sphere in CBCT images for visual registration with respective contours (Fig. 2). The large density difference between PMMA ($1.19g/{\text{cm}}^{3}$) and the surrounding polystyrene foam ($0.032g/{\text{cm}}^{3}$) renders clear images that are not affected by artifacts caused by the tungsten carbide ball. The coordinates of the center of the PMMA sphere are derived from the shift distances required for registration with reference images, displayed with 0.1 mm resolution on the console of the Varian STx accelerator. A similar procedure is used to register planar kV and MV images of the $15g/{\text{cm}}^{3}$ dense tungsten carbide ball with corresponding 6 mm diameter circles. For Winston‐Lutz (WL) tests, planar MV images of a MLC‐defined $1.5\times 1.5\;{\text{cm}}^{2}$ radiation field are registered with corresponding $1.5\times 1.5\;{\text{cm}}^{2}$ computer‐generated squares (Fig. 3). The difference between the shift vectors required for registration of the tungsten carbide ball compared to the shift vector for registering the field outline is a measure of targeting accuracy.

The outline of the 25 mm diameter sphere used as reference in CBCT registration was created in MATLAB (Version 8.3.0, MathWorks, Natick, MA) and imported into the treatment planning system (Eclipse version 11, Varian Medical Systems, Palo Alto, CA) via DICOM RT. The 6 mm diameter circle for registration of the tungsten carbide ball in planar kV and MV images was generated using the Eclipse contouring tools. Similarly, square outlines measuring $1.5\times 1.5\;{\text{cm}}^{2}$ were generated for WL tests of the MLC‐generated fields. All reference contours were attached to a treatment plan, like organ or PTV contours, of a patient and exported to the accelerator. We used computer‐generated contours because of their mathematical perfection. Reference contours from diagnostic CT scans could be encumbered by inaccuracies of the scans and cause systematic errors in subsequent QA tests.

## III. RESULTS

### A. Measurement accuracy of the UAB

The UAB was mounted on a 3D micrometer‐adjustable translation stage (Newport, Irvine, CA), positioned approximately at the isocenter of a Varian STx accelerator, and a series of CBCT images was taken (full rotation scan, 100 kV tube voltage, 270 mAs, $0.8\times 0.8\;{\text{mm}}^{2}$ pixels, 2.0 mm slice thickness). Between the individual CT scans, the UAB was shifted with the aid of the translation stage by distances known to within 0.01 mm. The shift distances were random, but confined to ±2 mm from the original position. Based on the CT images, six experimenters used the Offline Review tool of the Aria suite to find the distances along the three major axes by which the UAB had been repositioned. The discrepancy between measured and actual shifts was considered as the measurement error. The shift vector error *v* was computed as

(1) $$v=\sqrt{{\left(\Delta Vrt\right)}^{2}+{\left(\Delta Lng\right)}^{2}+{\left(\Delta Lat\right)}^{2}}$$

where ΔVrt, ΔLng, and ΔLat are, respectively, the measurement errors of the shifts along the vertical, longitudinal, and lateral directions. The error, averaged over all measurements made by an individual experimenter, is a measure of the uncertainty with which he/she found the position of the sphere in CBCT images. The error averaged over all experimenters was considered as a gauge of accuracy achievable with the UAB. Table 1 summarizes the results.

A similar procedure was used to assess the accuracy of finding the coordinates of the UAB in planar MV images (6 MV standard flattened beam, 3 MU) using the tungsten carbide ball as pointer. The imaging panel measured $40\times 30\;{\text{cm}}^{2}$, had a matrix of 1024×768 image receptors, and was positioned 50 cm below isocenter. Table 2 is a summary the results.

**Table 1:** Measurement errors and standard deviations (in mm) committed by six experimenters in determining shift distances of the UAB based on CBCT images. Data for each experimenter are averages over 14 shifts

*Exp*	ΔVrt	ΔLng	ΔLat	*v*	$v_{\text{max}}$
1	0.00±0.07	0.00±0.05	0.00±0.08	0.10±0.04	0.16
2	0.00±0.06	‐0.01±0.10	0.00±0.06	0.12±0.04	0.19
3	0.00±0.07	0.00±0.07	‐0.01±0.07	0.10±0.05	0.19
4	0.00±0.06	0.00±0.08	‐0.01±0.07	0.11±0.05	0.18
5	‐0.01±0.04	‐0.01±0.05	0.00±0.06	0.08±0.03	0.14
6	0.00±0.07	0.00±0.06	0.00±0.08	0.10±0.05	0.19
All	‐0.00±0.06	‐0.00±0.07	‐0.00±0.07	0.10±0.05	0.19

**Table 2:** Measurement errors and standard deviations (SD) (in mm) committed by six experimenters in determining shift distances of the UAB based on 2D MV images. Data for each experimenter are averages over 16 shifts

*Exp*	ΔLng	ΔLat	*v*	$v_{\text{max}}$
1	0.01±0.06	0.01±0.05	0.07±0.04	0.14
2	0.01±0.06	0.01±0.04	0.07±0.04	0.13
3	0.01±0.06	0.01±0.05	0.08±0.04	0.13
4	0.01±0.06	0.01±0.04	0.07±0.04	0.13
5	0.01±0.06	0.01±0.04	0.06±0.04	0.13
6	0.01±0.04	0.01±0.04	0.05±0.04	0.13
All	0.01±0.06	0.01±0.05	0.06±0.04	0.14

### B. Daily clinical QA of kV/MV image congruence, laser alignment, couch movement accuracy, WL tests

These tests are done by therapists during machine warmup and take about 10 min. The UAB is placed on the couch, aligned with the wall lasers to the offset marks (purposely offset by ‐3,7, and 5 mm along the vertical, longitudinal, and lateral directions, respectively), and anterior and lateral kV setup images are taken. By registering the images of the tungsten carbide ball with the corresponding computer‐generated circles, the offset distance of the ball from kV isocenter is measured and compared to the intentional offset. A discrepancy of >1 mm between measured and planned offset would indicate poor laser alignment and/or substantially inaccurate couch motion. After a couch shift to align the UAB with the kV isocenter, a CBCT is taken and registered with reference circles in the three principal planes. The shift distances required to move the PMMA ball to CBCT isocenter are read on the computer screen, recorded, and applied (Table 3). The small shift distances indicate that couch motions were accurate and that kV and CBCT isocenters coincided. It also shows the high accuracy achieved in routine clinical QA tests. A disagreement of >0.3 mm would indicate a misalignment of the imaging systems, inaccurate couch travel, and/or a defective UAB, and would trigger an investigation.

With the UAB positioned at CBCT isocenter, WL tests were done. Whereas traditional WL tests measure the distance between mechanical isocenter and beam axes defined by a circular tertiary collimator,^(14)^ our WL tests measure the distance between CBCT isocenter and central axes of MLC‐defined radiation fields. Considering that patients are set up according to CBCT images and that our SRS radiation fields are defined by MLCs, we believe that CBCT and MV isocenter agreement is one of the most important parameters to verify. For the WL tests, the MLC is set to $1.5\times 1.5\;{\text{cm}}^{2}$, the X‐ray jaws to $3\times 3\;{\text{cm}}^{2}$, and anterior and lateral MV images are taken with 6 MV beams at 3 monitor units (MU). The image of the tungsten carbide ball is then registered with the corresponding reference contour. The shift vector required for registration represents the discrepancy between the CBCT and the MV coordinate systems (Table 4, second column). Thereafter, the borders of the MLC‐defined field are shifted for registration with the corresponding computer‐generated square (Fig. 3), yielding the position of the central ray (CR) in the MV coordinate system (Table 4, third column). The difference between the two shift vectors is the vector by which the MLC‐defined CR misses CBCT isocenter (Table 4, last column). In the IEC 61217 coordinate system used by the STx accelerator, the positive lateral (X), longitudinal (Y), and vertical (Z) coordinate directions are oriented, respectively, toward the right, toward the gantry, and toward the ceiling as seen by and observer standing at the foot of the couch and facing the gantry. Note the shift of 0.245 mm in the longitudinal coordinate toward the foot of the couch as the gantry is rotated from the anterior to the lateral direction. In a traditional WL test, the data in the last column would correspond to the offset of the circular aperture with respect to the pointer at mechanical isocenter.

**Table 3:** Distance (in mm) required to shift the UAB from isocenter defined by planar kV images to isocenter defined by CBCT images. Shift distances were obtained by visual match of CBCT images of the PMMA sphere to computer‐generated circles in the cross‐sectional, coronal, and sagittal planes. Data represent averages of 383 daily morning QA measurements on a STx accelerator

*Direction*	*Shift Distance*
Vrt (Z)	‐0.02±0.10
Lng (Y)	0.01±0.08
Lat (X)	0.09±0.12
v	0.16±0.12

**Table 4:** Winston Lutz tests by therapists as part of machine warm‐up, averaged over 383 treatment days. Second column: misalignment between CBCT and MV coordinates (in mm). Last column: distance by which the central ray of the MLC‐defined field misses CBCT isocenter. The differences in the Y coordinates between anterior and lateral fields are due to gantry flex

*Coordinate*	*Coordinates of MV Isocenter in CBCT Coordinate System*	*Coordinates of Central Ray in MV Coordinate System*	*Coordinates of Central Ray in CBCT Coordinate System*
Vrt (Z)^‡^	0.11±0.12	‐0.11±0.08	0.00±0.11
Lng (Y)^†^	0.13±0.17	‐0.03±0.20	0.10±0.19
Lng (Y)^‡^	0.06±0.19	‐0.21±0.18	‐0.14±0.15
Lat (X)^†^	‐0.04±0.13	0.07±0.08	0.03±0.14

a ^†^ Coordinates extracted from AP image.

b ^‡^ Coordinates extracted from LAT image.

## IV. DISCUSSION

The precision in finding CBCT and MV isocenter offered by the UAB in conjunction with the visual registration method may be even better than the experiments suggest. Offline review provided readout of shift distances with only 0.1 mm resolution, whereas much smaller shift distances were discernible on the computer screen during image registration. Furthermore, when shift distances were extracted from consecutive CBCT scans, any inaccuracy in the imaging chain would have added to the observed errors and erroneously degraded the accuracy of the QA method.

The morning QA checks, being end‐to‐end verifications of essential machine parameters with sub‐mm accuracy, gave confidence, especially on days following evening machine maintenance, that SRS and SBRT could be accurately delivered. Narrow display windows at the proper density level yield sharp outlines of the spheres and the MLC fields that can be readily and precisely matched with computer‐generated contours having equal shape and size. Since the QA system does not use manufacturer‐supplied hardware or software, it constitutes a truly independent check of machine performance. Computer assistance could provide even more accuracy and speed up the QA process, but the visual registration of the spherical markers is done quickly and provides a human touch that is intuitive and transparent to the operator.

The UAB was made from off‐the‐shelf components and can be duplicated by any suitable machine shop. The critical centering of the PMMA and tungsten carbide sphere was achieved by an experienced machinist operating a milling machine with digital position readout having 0.01 mm resolution. The acrylic sphere was held by a collet block of equal diameter that was aligned with the spindle axis of the milling machine using a dial gage. A 6.35 mm ball‐end mill was used to drill a hole to the required depth. The depth was measured by placing the 6.35 mm tungsten carbide ball into the hole and, using a micrometer, recording the distance from the proximal surface of the metal ball to the distal surface of the PMMA ball. A small piece of a 0.02 mm–thick plastic foil had to be inserted at the bottom of the hole as a shim to correct for a slightly excessive depth. The tungsten carbide ball was secured in its positon by a drop of adhesive. Considering the precision of the manufacturing tools and process, the concentricity of the balls is estimated as within 0.05 mm.

The outer shell of the UAB was machined from a block of PMMA. Following careful alignment of the block along the spindle axis of the milling machine, a boring head was used to drill a 65 mm diameter cylindrical cavity. While keeping the block in the chuck of the milling machine, a firmly fitting cylindrical polystyrene foam plug was inserted into the cavity. A 2.54 mm diameter ball‐end mill running at low speed and slow advance generated a smooth hole for the concentric spheres. The spheres were secured in place by a polystyrene foam plug and a PMMA face plate. Their correct position was verified by a depth gauge inserted through an opening in the face plate (Fig. 1), estimated to agree within 0.1 mm with the scribe marks on the surface of the UAB provided for laser alignment.

## V. CONCLUSIONS

The UAB phantom in conjunction with visual registration provides sufficient accuracy for assuring the level of accelerator performance required for even the most demanding SRS and SBRT treatments. Judicial selection of a narrow image display window at the appropriate level greatly enhances the speed and accuracy of the registration process. The QA method is practical for routine morning checks by therapists. The STx accelerator together with the imaging system and the MLC consistently provides sub‐mm accuracy.

## ACKNOWLEDGMENTS

The authors are grateful to Mr. Jerry Sewell from the Department of Physics for precision‐manufacturing many of the components of the UAB test device and for his technical support, and to Mr. Rex Bennett for many fruitful discussions. We are also thankful to our dedicated RTT team, Brandon Beard, Danielle Bentley, Leesa Cordell, Nathan Jordan, Karen Kirksey, LaKeisha Moore, Debra Myers, Chrystie Myers, Kim Poulos, Freddie Ray, Jason Schneider, and Shashank Singh, whose daily morning QA tests were essential for establishing accuracy and practicality of the method.

## COPYRIGHT

This work is licensed under a Creative Commons Attribution 3.0 Unported License.
